# Frailty and lived experiences in older adults with osteoporosis: protocol for a convergent mixed-methods study

**DOI:** 10.3389/fmed.2025.1683445

**Published:** 2026-01-20

**Authors:** Jinli Wang, Xinling Gao, Shuang Li, Mei Lin, Yaqi Lan, Zhen Tan, Pei Liu

**Affiliations:** 1 Health Science Center, Yangtze University, Jingzhou, Hubei, China; 2 Peking University Shenzhen Hospital, Shenzhen, Guangdong, China; 3 School of Nursing, Anhui Medical University, Hefei, Anhui, China; 4 Shantou University Medical College, Shantou, Guangdong, China; 5 School of Nursing and Health, Henan University, Kaifeng, Henan, China

**Keywords:** older adults, osteoporosis, frailty, mixed-methods study, protocol

## Abstract

**Background:**

Older adults is characterized by an age-related decline in bone mineral density. Frailty-a distinct age-associated geriatric syndrome-further exacerbates this condition. The concomitance of osteoporosis and frailty in older adults markedly heightens the risk of functional decline, with attendant profound psychosocial sequelae. A mixed-methods study design is essential to achieve a comprehensive, patient-centered understanding of this phenomenon.

**Methods:**

This study will adopt a convergent mixed-methods design comprising three primary phases: (1) Quantitative phase: a cross-sectional survey will be conducted among older adults diagnosed with osteoporosis. Data collection will include demographic variables, scores from the Edmonton Frail Scale, Activities of Daily Living assessments, Nutritional Risk Screening assessments, and the Hospital Anxiety and Depression Scale. In addition, handgrip strength will be measured, alongside relevant laboratory parameters. Data will undergo descriptive analysis, followed by univariate analyses and multivariate binary logistic regression to identify factors associated with frailty. (2) Qualitative phase: semi-structured, face-to-face interviews will be performed with participants classified as frail according to their frailty assessment scores, enabling an in-depth exploration of their subjective perceptions and experiences of frailty within the context of osteoporosis. (3) Integration phase: key findings from both quantitative and qualitative components will be analyzed and synthesized to generate a comprehensive understanding of the phenomenon.

**Discussion:**

This study aims to explore the prevalence and clinical characteristics of frailty among older adults with osteoporosis. It also seeks to identify associated risk factors and to explore patients' subjective experiences of living with frailty in the context of osteoporosis.

**Clinical trial registration:**

This trial has been registered with the Chinese Clinical Trial Registry (ChiCTR2500097059).

## Introduction

Osteoporosis (OP) is a systemic skeletal disorder characterized by reduced bone mineral density and microarchitectural deterioration of bone tissue (1). It is highly prevalent among older adults and, owing to its insidious onset and the potentially devastating consequences of fragility fractures, is a major global public health concern. This condition substantially compromises functional independence, contributing to overall functional decline in the aging population (2). A meta-analysis published in 2021 estimated the global prevalence of osteoporosis among older adults at 21.7%, with a higher prevalence of 24.3% reported in older Asian populations (3). Osteoporosis markedly increases the risk of fragility fractures. According to the IOF, one-third of women and one-fifth of men aged ≥50 years will experience a fracture. These events lead to reduced quality of life, higher hospitalization rates, long-term disability, and increased mortality (4, 5). In China, the growing burden of osteoporosis and related fractures is being exacerbated by the rapidly aging population. Recent meta-analyses show a clear age-related increase in prevalence, with rates escalating significantly after age 60 (6). Furthermore, the functional consequences of fractures are severe: approximately 20–30% of patients fail to regain their pre-fracture functional status, experiencing increased dependency and a significantly increased risk of mortality (7).

Frailty is an age-associated geriatric syndrome that represents a critical intermediate state in the aging trajectory. Its defining feature is a decline in physiological reserve, resulting in markedly reduced resilience to both endogenous and exogenous stressors (8). This decline is underpinned by measurable biological processes across multiple domains. Consensus biomarkers for frailty highlight systemic involvement, specifically encompassing the physiological domain (e.g., insulin-like growth factor 1), the inflammatory domain (e.g., high sensitivity C-reactive protein, interleukin-6), and the epigenetic domain (e.g., DNA methylation/epigenetic clocks) (9). Furthermore, frailty extends beyond these physiological deficits to a multidimensional syndrome that severely compromises functional independence (as measured by muscle mass and handgrip strength) and encompasses psychosocial domains, including social support and psychological well-being (10). The clinical significance of this complex syndrome is evidenced by its high prevalence, with epidemiological studies reporting rates reaching 29.8% in hospital settings, and pre-frailty observed in an additional 39.3% of patients (11).

A meta-analysis published in 2025 reported that the prevalence of osteoporosis concomitant with frailty among older adults reached 37.8%. This comorbidity results in physical functional impairment and often precipitates profound psychosocial consequences, including fear of falling, reduced mobility, diminished social participation, symptoms of depression and anxiety, altered self-perception, increased dependency, contributing to an overall psychosocial burden and functional decline (12). This indicates that over one-third of older adults with osteoporosis are also frail, underscoring the urgent need for integrated management approaches. Furthermore, evidence is accumulating to clarify the intricate mechanistic link between these two geriatric syndromes. A recent study utilizing the National Health and Nutrition Examination Survey (NHANES) for a cross-sectional analysis and Mendelian randomization (MR) not only confirmed a significant positive correlation but also established a bidirectional causal relationship between osteoporosis and frailty. Building on this, a prospective study provided crucial mechanistic clarity, demonstrating that chronic low-grade systemic inflammation (indicated by biomarkers like the Neutrophil-to-Lymphocyte Ratio and Systemic Immune-Inflammation Index) acts as an upstream driver. Critically, the frailty phenotype itself partially mediates the association between this systemic inflammation and the resulting increase in osteoporosis and fracture risks (13). These interconnected factors, driven by common pathways including sarcopenia and endocrine dysregulation, collectively amplify the susceptibility to falls and fractures, thereby constituting a significant functional risk for older adults with osteoporosis (14). Although the coexistence of frailty and osteoporosis has been studied epidemiologically, few studies have explored the subjective lived experiences in this population using a mixed-methods design.

This study offers a unique contribution by being among the first to utilize a convergent mixed-methods design for an in-depth exploration of the frailty experience in older adults with osteoporosis within the Chinese context. Most frailty-related research to date has predominantly focused on community-dwelling older adults and patients with chronic conditions such as cardiovascular disease, chronic kidney disease, diabetes mellitus, and cancer (15–17). Research on frailty among patients with osteoporosis remains limited, primarily addressing risk factors and often restricted to a single physiological domain. Notably, there is a paucity of in-depth qualitative investigation into how patients with osteoporosis perceive and understand frailty—specifically, their lived illness experience. Illness experience encompasses patients' perceptions, interpretations, emotional responses, disruptions to daily life trajectories, coping mechanisms, and interactions with healthcare services. Although standardized assessment instruments provide valuable quantitative data, they frequently fail to capture the richness, complexity, and individual variability inherent in the illness experience. Consequently, these tools are insufficient to elucidate the underlying socio-cultural, psychological, and environmental determinants reflected in the scores (18).

Therefore, there is an urgent need for a comprehensive study to characterize the current disease status of older adults with osteoporosis and to explore their lived illness experience in depth. Such research will provide an essential foundation for the development of more precise and patient-centered intervention strategies. To address this research gap, the present study will employ a concurrent convergent mixed-methods design. This methodology facilitates a holistic understanding of frailty in older adults with osteoporosis through a threefold integrative approach that combines quantitative and qualitative data (19). Ultimately, by integrating these two perspectives, this study moves beyond the quantification of risk factors to explore the subjective lived experiences of frailty—a crucial step for developing holistic, patient-centered interventions.

This study aims to: (1) determine the prevalence and current status of frailty among older adults with osteoporosis; (2) identify factors associated with frailty within this population; (3) explore the subjective illness experiences of frailty in older adults with osteoporosis; (4) analyse potential discrepancies between healthcare system frailty management recommendations and patients' illness perceptions; and (5) elucidate conflicting mechanisms between qualitative and quantitative findings through triangulation analysis. By addressing these objectives, this research seeks to provide evidence-based insights to inform the development of precise, patient-centered nursing care and health policy.

## Materials and methods

### Study design

**Figure 1:**
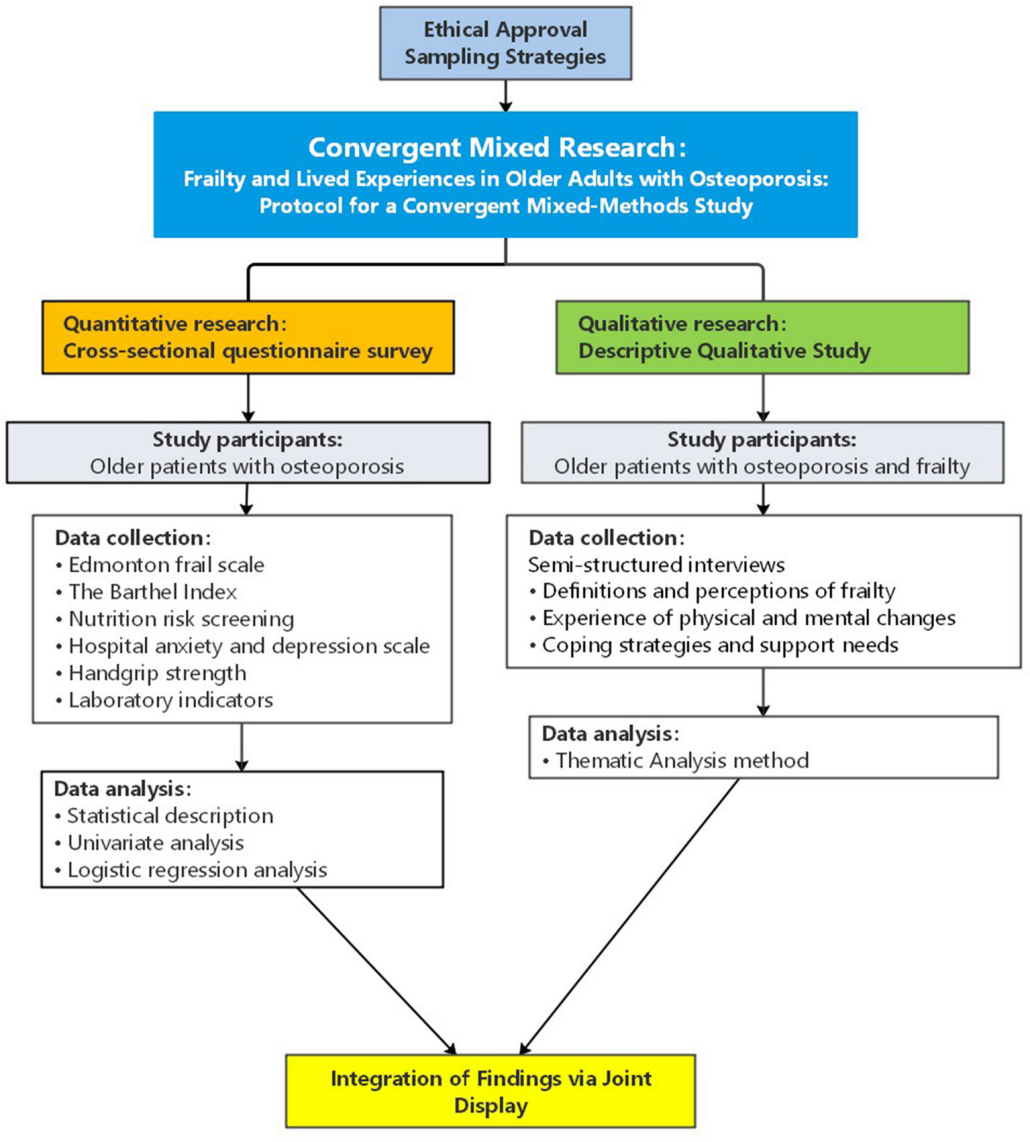
Flowchart of the convergent mixed-methods study design.

This study will employ a convergent mixed-methods approach, involving the concurrent collection of quantitative and qualitative data within the orthopedic wards of a tertiary hospital from February to December 2025. Data will be analyzed separately before integrating the results (see Figure 1).

The study will proceed through three sequential yet interrelated phases:

(1) Quantitative phase

- A cross-sectional survey will be administered to older adults diagnosed with osteoporosis.
- Data will include: demographic characteristics; Edmonton Frail Scale (EFS) scores; Activities of Daily Living (ADL) assessments; Nutritional Risk Screening (NRS) scores; Hospital Anxiety and Depression Scale (HADS) scores; handgrip strength measurements; and relevant laboratory parameters.
- Statistical analysis will involve descriptive statistics, univariate analyses, and multivariate binary logistic regression to identify factors associated with frailty.

(2) Qualitative phase

- Semi-structured, face-to-face interviews will be conducted with patients classified as frail based on their frailty scores.
- This component aims to provide an in-depth exploration of participants' lived experiences of frailty in the context of osteoporosis.

(3) Integration phase

- Principal findings from both datasets will be compared and synthesized.
- Integration will be achieved through joint display analysis and narrative synthesis, enabling the identification of convergent, complementary, or divergent findings.
- his phase is designed to yield novel insights and enhance clinical practice guidance for frailty management in osteoporotic patients.

### Ethics and registration

This study received ethical approval from the Medical Ethics Committee of Peking University Shenzhen Hospital, Beijing, China (approval number [2025] No. 003). It has also been registered with the Chinese Clinical Trial Registry (registration number ChiCTR2500097059). Prior to participation, all subjects will be fully informed of the study's purpose and significance and will provide written informed consent, in accordance with the Declaration of Helsinki.

### Stage1: quantitative study

For the quantitative component, this cross-sectional study will employ convenience sampling to recruit patients diagnosed with osteoporosis from a tertiary A-level hospital in Shenzhen, Guangdong Province, China. However, we acknowledge that convenience sampling may introduce selection bias, which could limit the representativeness of the sample and the generalizability of the findings.

#### Participants

Inclusion criteria are:

(1) Age ≥ 65 years;
(2) Osteoporosis Diagnosis: diagnosis of osteoporosis based on any of the following:

- a dual-energy *X*-ray absorptiometry (DXA) *T*-score of ≤ −2.5 at the axial skeleton or distal one-third radius;
- a history of fragility fracture of the hip or vertebra;
- osteopenia (−2.5 < *T*-score < −1.0) accompanied by a fragility fracture of the proximal humerus, pelvis, or distal forearm (20);

(3) Possessing good communication skills, the ability to read questionnaires effectively, and intact cognitive function (e.g., scoring ≥24 on the Mini-Mental State Examination [MMSE] threshold);
(4) voluntary participation with signed informed consent.

Exclusion criteria are:

(1) Patients with other critical or unstable illnesses, such as active malignant tumors or severe, acute cardiorespiratory insufficiency;
(2) Patients exhibiting severe disability, bedridden status, or conditions that severely limit mobility required for frailty assessments;
(3) A history of stroke or severe head trauma, or existing central nervous system diseases;
(4) Individuals unable to cooperate with questionnaire surveys or frailty assessments.

#### Sample size

Based on a previous meta-analysis reporting a frailty prevalence of 53.3% in older adults with osteoporosis, the required sample size was first calculated using a single-sample proportion estimation formula (21). With an anticipated proportion (*P*) of 0.533, an alpha (α) level of 0.05 (*Z* α/2 = 1.96), and an allowable error (*d*) set at 0.1 *P* = 0.0533, the initial calculation yielded an *N* of 343. Furthermore, for the planned multivariate logistic regression (using G^*^Power 3.1.9.7), we assumed a medium effect size (Odds Ratio = 1.6), α = 0.05, and statistical power of 0.80. This calculation indicated that a sufficient sample size for the regression analysis is less than the calculated 343. Considering a 10% non-response rate, the total sample size required is a minimum of 378 participants, which ensures adequate power for both prevalence estimation and subsequent regression analysis.

#### Instrument

Data will be collected using the following research instruments:

- The General Information Questionnaire will collect 17 variables: sex, age, height, weight, body mass index (BMI), marital status, living arrangements, educational attainment, monthly household income, medical expense reimbursement method, presence of insomnia, exercise habits, smoking status, alcohol consumption, calcium supplementation, history of falls within the previous 6 months, and 17 comorbid conditions.
- Edmonton Frail Scale (EFS): developed by Rolfson et al. in 2006, the Edmonton Frail Scale (EFS) provides a comprehensive assessment of frailty encompassing physical, psychological, and social domains (22). This 11-item instrument evaluates frailty across nine key domains: cognition, general health status, independence, social support, medication use, nutrition, mood, continence, and self-reported functional performance. Cognitive function is assessed using the Clock Drawing Test, while mobility is measured via the Timed Up and Go (TUG) test. Independence in activities of daily living is quantified by the number of independently completed tasks out of eight routine activities, including cooking, cleaning, and shopping. The EFS yields a total score ranging from 0 to 17, with higher scores indicating greater frailty severity. The scale is user-friendly, practical, and can be administered within approximately 10 min, making it suitable for rapid frailty screening by both healthcare professionals and trained non-professionals. Internal consistency is acceptable, with a reported Cronbach's alpha coefficient of 0.62.
- Activities of Daily Living Assessment Scale (ADL): the Barthel Index is a widely used instrument to evaluate activities of daily living in older adult patients. This scale comprises 10 items, including feeding, grooming, dressing, bowel control, bladder control, toilet use, transfers (bed to chair), ambulation on level surfaces, and stair climbing (23). The total score ranges from 0 to 100, with higher scores indicating greater independence in performing daily self-care tasks. Based on the total score, patients' functional status is classified into five categories: complete dependence (0–20 points), severe dependence (21–40 points), moderate dependence (41–60 points), mild dependence (61–99 points), and complete independence (100 points).
- Nutritional Risk Screening Assessment Scale: the Nutritional Risk Screening 2002 (NRS-2002) was developed through international collaboration among researchers from Denmark, the United Kingdom, France, and other countries. It is designed to evaluate changes in nutritional status based on parameters such as weight variation over the preceding 3 months and dietary intake during the past week (24). The scale demonstrates moderate reliability, with a Kappa coefficient (κ) of 0.67, a sensitivity of 0.86, and a specificity of 0.37.
- Hospital Anxiety and Depression Scale (HADS): developed by Zigmond et al. in 1983, the Hospital Anxiety and Depression Scale (HADS) is a self-administered instrument designed to evaluate anxiety and depression symptoms experienced by patients over the preceding week (25). It comprises two subscales—anxiety and depression—each containing seven items scored on a four-point Likert scale ranging from 0 to 3. The total score for each subscale ranges from 0 to 21. Scores are interpreted as follows: 0–7 denotes no clinically significant symptoms; 8–10 indicates possible presence of anxiety or depressive symptoms; and 11–21 reflects probable clinical anxiety or depression. The internal consistency reliability, expressed by Cronbach's α coefficients, is 0.879 for the total scale, and 0.806 for both the anxiety and depression subscales.
- Muscle Strength Assessment: muscle strength will be evaluated using a handheld dynamometer. Participants will be instructed to perform a maximal voluntary isometric contraction by gripping the dynamometer handle with their dominant hand for 2–3 s. Two measurements will be recorded, with the highest value (expressed in kilograms) retained as the final result, reported to two decimal places.
- Laboratory Indicators: within 24 h of admission, the following laboratory parameters will be collected: neutrophil percentage (%), hemoglobin (g/L), C-reactive protein (mg/L), serum calcium (mmol/L), and serum creatinine (μmol/L). Normal reference ranges are as follows: neutrophil percentage: 40–75%. Hemoglobin: 130–175 g/L for males, 115–150 g/L for females. C-reactive protein: 0–10 mg/L. Serum calcium: 2.11–2.52 mmol/L. Serum creatinine: 57–111 μmol/L for males, 41–81 μmol/L for females.

#### Date collection

Under the supervision of the research team, standardized training on study procedures will be provided. After explaining the study's purpose and significance and obtaining informed consent, data will be collected via one-to-one interviews. For inpatients, questionnaires will be administered following their daily treatments and once they report no significant discomfort. If participants are unable to complete the questionnaire independently, the investigator will record responses based on participants' verbal reports.

Demographic data, Edmonton Frail Scale (EFS) scores, Activities of Daily Living (ADL) assessments, Nutritional Risk Screening (NRS) assessments, Hospital Anxiety and Depression Scale (HADS) scores, and muscle strength measurements will be collected through face-to-face interviews. Laboratory parameters will be retrieved from the electronic medical records system. Each questionnaire is estimated to require 15–20 min for completion. All questionnaires will be administered and collected on-site.

Following the survey, investigators will offer psychological support to participants to mitigate any distress experienced during questionnaire completion. Throughout the study, the ethical principles of autonomy, confidentiality, and non-maleficence will be rigorously upheld. Finally, all assessment results will be independently reviewed by two qualified medical professionals with a minimum of intermediate-level clinical credentials to reduce potential bias.

#### Data analysis

All collected data will undergo quality control prior to statistical analysis using SPSS version 27. Continuous variables with a normal distribution will be presented as mean ± standard deviation, and intergroup comparisons will be conducted using independent samples *t*-tests. Continuous variables not normally distributed will be summarized as median and interquartile range, with between-group comparisons performed using the Mann–Whitney U test. Categorical variables will be expressed as frequencies and percentages, and comparisons analyzed via chi-squared tests. Ordinal data will be assessed using non-parametric rank-sum tests. A two-sided significance level of *p* < 0.05 will be adopted for all statistical tests. Variables demonstrating *p* < 0.05 in univariate analysis, along with clinically relevant covariates identified through consultation with clinical experts, will be entered into the multivariate logistic regression model.

### Stage2: qualitative study

This study will adopt a descriptive qualitative methodology, employing face-to-face interviews with older adults diagnosed with osteoporosis and concomitant frailty to explore their perceptions and lived experiences of frailty.

#### Participants

Following purposive sampling principles, a representative sample will be selected based on factors including age, educational attainment, and frailty severity (26). Participants identified as frail according to the Edmonton Frail Scale will be recruited for semi-structured, face-to-face interviews. All participants will provide informed consent and voluntarily agree to participate in the study.

#### Sample size

The final sample size will be determined by data saturation. Approximately 15–20 frail participants will be interviewed until thematic saturation is achieved. Recruitment will cease once no new themes emerge and participant responses become repetitive during analysis.

#### Instrument

The primary research instrument is administered by an investigator who has undergone systematic training in qualitative research and conducted comprehensive literature reviews, actively participating in research team discussions. The interview guide was initially developed based on Rockwood's Frailty Assessment Theoretical Model and the Multidimensional Frailty Model (27, 28). Rockwood's Frailty Index (FI), a cumulative deficit model, conceptualizes frailty as the accumulation of multisystem, multidimensional deficits. In this interview framework, the FI model is primarily applied to indirectly capture the progressive accumulation of patient deficits associated with aging.

The Multidimensional Frailty Model conceptualizes frailty as a complex, multifactorial syndrome that extends beyond physiological decline to encompass psychological, social, and cognitive dimensions. This broader framework informs the interview process, allowing for a comprehensive exploration of older adults' lived experiences of frailty across various life domains. Semi-structured interviews will begin with the following core questions: (1) “How do you define frailty?” (2) “What physical and psychological changes have you experienced as you've aged?” (3) “What changes have occurred in your daily life as you've grown older?” (4) “How do you cope with or address these issues?” (5) “What kind of support or assistance do you require during these processes?” These questions will be iteratively refined and adapted throughout the interview process in response to emerging themes.

#### Data collection

This study will adopt a descriptive qualitative research design, utilizing one-on-one, face-to-face semi-structured interviews to elicit participants' lived experiences. Each interview will last approximately 30–40 min and will be conducted in a quiet, private teaching room within the department to minimize external disturbances. Ethical principles of informed consent, voluntariness, and confidentiality will be strictly upheld. Prior to each interview, the researcher will introduce themselves and clearly explain the study's purpose, estimated duration, and the rationale for audio recording. Upon obtaining informed consent, interviews will be audio-recorded, and concurrent field notes will be taken. During the interviews, the researcher will adopt a neutral, non-directive stance, avoiding any leading questions or judgmental language. Clarification will be sought using non-suggestive probing where ambiguity arises. Non-verbal cues—including vocal tone, intonation, facial expressions, and body language—will also be documented to ensure rich and accurate data collection. All audio recordings will be transcribed verbatim within 24 h of the interview, followed by a secondary verification of the transcript. To proactively manage researcher bias and ensure objectivity (reflexivity), researchers will maintain reflective journals throughout the data collection and analysis phases to document their preconceptions and assumptions. Transcribed data will subsequently be returned to participants for member checking to confirm accuracy and enhance credibility.

#### Date analysis

Qualitative data will be analyzed using Thematic Analysis (such as the six-phase method by Braun and Clarke), supported by NVivo 11 software for systematic coding and data management. This approach aims to thoroughly analyze the patterns and themes within the subjective experiences of frailty among older adults with osteoporosis (29).

### Stage3: integration study

Quantitative and qualitative data will be analyzed independently, followed by systematic integration using a joint display approach. In the results and discussion sections, key quantitative findings will be presented alongside principal qualitative themes through the creation of matrix tables (joint displays) and figures. This visualization will serve as the primary method for synthesis, allowing for a direct comparison and combined interpretation of the two data types using narrative description.

The analysis during this integration process will systematically focus on:

- Direct Comparison and Convergence: we will use matrix tables to display statistical results (e.g., associated factors) alongside the supporting qualitative themes and illustrative quotes. Convergence is established when themes directly confirm quantitative findings.
- Elaboration and Contextual Explanation: qualitative data will be utilized to interpret ‘how' and ‘why' a quantitative finding occurs (e.g., patient quotes will explain the mechanisms underpinning a specific statistical association).
- Discrepancy Exploration: divergences (contradictory findings) will be treated as an important source of insight. We will investigate these conflicts by re-examining the quantitative outliers in light of the qualitative context to reveal unmeasured conceptual factors or sampling differences.

Ultimately, this integration will generate a comprehensive, holistic understanding of frailty and the illness experience in older adults with osteoporosis, enhancing the depth and utility of the study findings.

## Discussion

Frailty in older adults with osteoporosis is not merely a physiological burden; it is a multidimensional geriatric syndrome that profoundly affects their well-being and overall illness experience. While existing research has primarily focused on quantifiable biological parameters, it often overlooks patients' subjective perceptions and lived experiences of frailty (30).

This study adopts an innovative mixed-methods research design, which we consider a major strength. Unlike prior studies that focused largely on biomedical parameters, this design uniquely integrates quantitative and qualitative perspectives to capture patients' lived experiences and socio-cultural contexts. Through the analysis of multiple dimensions—including physical, psychological, and nutritional factors—this research aims to comprehensively elucidate the current status and multifaceted impact of frailty in older adults with osteoporosis.

Despite its strengths, we proactively acknowledge several limitations: (1) Generalizability: the use of a single recruitment site may limit the representativeness of the sample, thereby reducing the generalizability of the findings to broader populations of older adults. (2) Cross-Sectional Design: the quantitative component is cross-sectional in design, which restricts causal inference and allows only the identification of associations. Nonetheless, it is anticipated that this component will yield meaningful insights and serve as a foundation for future multi-center or longitudinal investigations. (3) Data and Articulation Bias: older participants may have difficulty articulating their experiences clearly during qualitative interviews, and there may be comprehension bias in completing quantitative questionnaires. Mitigation: to minimize these issues, all interviewers will undergo standardized training, employ clear and accessible language, thoroughly explain all questionnaire and interview items, and provide assistance as needed. (4)Methodological Complexity: integrating qualitative and quantitative data presents inherent methodological challenges. Mitigation: to address this, a detailed integration protocol has been developed from the outset, all team members will receive training in mixed-methods research, and the involvement of experienced mixed-methodologists will be considered for guidance and consultation.

The results of this study are expected to serve as a bridge between research evidence and clinical practice, offering guidance for the design of targeted interventions and the improvement of patient-clinician communication. Specifically, the findings, supported by existing literature demonstrating a strong association between frailty and vertebral compression fractures (VCFs), may contribute to the development of frailty-specific care pathways in osteoporosis management (31). At the clinical practice level, these discoveries provide a basis for establishing tailored rehabilitation programs, effective fall-prevention strategies, and needs-based nutritional interventions. For instance, by elucidating the potential complex interplay among diet-related quality of life (DRQOL), protein intake, and frailty, this research offers preliminary evidence to integrate strategies aimed at improving DRQOL and protein status, moving beyond traditional bone density-focused dietary advice. Such strategies may potentially help mitigate the progression of frailty (32). Furthermore, given the documented association between frailty and a higher incidence of multiple fractures, the study's outcomes support the necessity of routine frailty screening in all older adults with osteoporosis. On a policy level, these results can inform geriatric health strategies aimed at reducing fracture risk, functional decline, and the escalating healthcare burden associated with the bidirectional relationship between frailty and osteoporotic fractures. Ultimately, this research will contribute empirical data to support integrated management strategies and enhance the quality of life for older populations.

Furthermore, this work serves as a foundational step toward more in-depth future investigations. The successful identification of key influencing factors will provide a valuable, evidence-based foundation for wider application across diverse geographical settings and patient populations. The anticipated results will inform the development and implementation of targeted interventions, offering robust empirical evidence to support clinical practice and health policy formulation. Ultimately, this research has the potential to enhance healthcare delivery, improve quality of life for older adults with osteoporosis, and reduce frailty-related complications at both the clinical and population levels.
